# Patient and physician preferences for non-invasive diagnostic cardiovascular imaging technologies: a discrete choice experiment

**DOI:** 10.1186/s41687-022-00419-0

**Published:** 2022-02-19

**Authors:** Thomas Bertrand, Gillian Bartlett-Esquilant, Kady Fischer, Matthias G. Friedrich

**Affiliations:** 1 Department of Family Medicine, McGill University, 5858 Côte-des-Neiges Road, Montreal, QC H3S 1Z1 Canada; 2 Departments of Medicine and Diagnostic Radiology, Research Institute of the McGill University Health Center, 1001 Decarie Boulevard, Montreal, QC H4A 3J1 Canada

## Abstract

**Purpose:**

Diagnostic imaging techniques have to be selected for their accuracy, efficiency, cost-efficiency, and impact on outcome. But beyond that, the choice of non-invasive cardiovascular imaging tests for diagnosing coronary artery disease also has to respect patient safety and comfort. In this study, we investigated patient and physician preference in relation to the choice of cardiovascular imaging tests.

**Results:**

A total of 211 subjects (148 cardiac patients and 63 physicians) were enrolled and completed a discrete choice experiment. Tests and modalities were deconstructed into 6 attributes (*risks and side effects*, *diagnostic accuracy*, *patient out-of-pocket cost*, *type of procedure*, *type of scanner* and *test duration*). A Sawtooth software choice-based conjoint analysis with hierarchical Bayes estimation was performed and showed the *risks and side effects* attribute was assigned the most relative importance (30%) when considering patients’ preference. Patients gave notably high value to tests with milder side effects, while preferring to avoid exposure to ionizing radiation and risks associated the use of pharmacological agents inducing direct coronary arteriolar vasodilation. Physicians allocated more importance to the *patient out-of-pocket cost* attribute (29%). Both patients and physicians valued tests’ *risks and side effects*, *diagnostic accuracy*, *patient out-of-pocket cost* as the three most important attributes, but in diverging order. A market simulation comparing current cardiovascular imaging tests revealed breathing maneuver-enhanced cardiac magnetic resonance had the highest shares of preference in both patients (59.6%) and physicians (32.7%).

**Conclusion:**

A patients’ preference for a particular cardiovascular imaging test was most determined by the risks and side effects, while physicians prioritized less costly tests for their patients. In shared decision-making with patients, physicians should therefore focus on a balanced discussion of risks and side effects associated with cardiovascular imaging tests. Both, patients and physicians would prefer a cardiovascular MR imaging test using a vasoactive breathing maneuver instead of currently used alternatives that require intravenous contrast agents, pharmacological stress, or radiation.

## Introduction

The diagnostic examination of suspected coronary artery disease (CAD) can involve a variety of tests and procedures. Each of these tests bear advantages and limitations, and considering none are vastly superior to the others, the American Heart Association and the Canadian Cardiovascular Society documented the importance of patient preference research to guide clinical decision-making and committed to develop a patient-centered approach to cardiovascular care [1–3]. Their recommendations emphasized the role of enhanced clinician–patient communications, and focused medical education and training related to patient-centered communication. This is especially important considering most of the technological breakthroughs in diagnostic cardiology have been driven to improve diagnostic accuracy [4], with little attention devoted to patient-centered outcomes such as safety, comfort, and overall satisfaction, with the latter being a central aspect of patient-centered care [5]. Patient-centered care, or care that is respectful of and responsive to individual patient preferences, has been described as 1 of 6 domains of clinical care quality, along with safety, effectiveness, timeliness, efficiency, and equity [6]. Enriched clinician–patient communication and shared decision-making require that physicians remain attentive to what is meaningful and valuable to the patient while recognizing that their initial belief may not align with their patient’s preference [7–9]. Although time constraints are a common challenge to patient-centered care [10], discussions to determine individual patient preference are important before creating an appropriate management plan.

In this context, the purpose of this study was to contribute to the understanding of patient and physician preferences in relation to the choice of advanced non-invasive cardiovascular tests and imaging modalities used for diagnosing CAD. Specifically, we aimed to answer the following research question: What characteristics of advanced non-invasive cardiovascular imaging tests are most important to patients likely to undergo imaging to characterize coronary stenosis, and to physicians likely to order a cardiac imaging test for their patient?

## Methods

### Study design

A discrete choice experiment (DCE) was designed to assess cardiac patients’ and physicians’ (cardiologists and family physicians) preference toward non-invasive cardiovascular diagnostic imaging tests. This study design employs a conjoint analysis survey method commonly used to elicit preferences by measuring the relative value of competing health-related services without requiring participants to personally experience these services; an advantageous rationale when studying costly and sometimes risky healthcare interventions such as cardiovascular imaging [11]. The DCE consists of presenting participants with samples of distinctive hypothetical service options, such as non-invasive cardiovascular imaging tests, from which they are invited to choose the one they prefer. By presenting participants with multiple test alternatives and inviting them to choose the alternative they prefer multiple times within a one survey, it is possible to estimate with good predictive value what characteristics, or attributes, of the tests influence their choice and to evaluate the relative preference they allocate to different levels of these attributes [12].

This study acknowledged the International Society for Pharmacoeconomics and Outcomes Research (ISPOR) guidelines for DCEs and conjoint analysis applications in health [13].

### Identifying attributes and levels

A review of the existing literature and semi-structured interviews with cardiac patients and cardiologist was performed to identify core attributes and levels for the DCE. Seven distinctive non-invasive cardiovascular imaging tests for diagnosing CAD were identified: Exercise stress echocardiography (echo), pharmacological stress echo, computed tomography (CT) angiography, cardiac positron emission tomography (PET), cardiac single photon emission computed tomography (SPECT), stress perfusion cardiac magnetic resonance (CMR), and, as a novel approach, breathing maneuver-enhanced CMR (b-CMR). These exams were subsequently deconstructed into 6 core attributes, to which each was assigned applicable levels (Table 1). While each imaging tests considered in this study have unique properties, tests’ attributes were grouped into inclusive levels.

**Table 1:** DCE attributes and levels

Attributes	Levels
Type of procedure	Exercising; pharmacological agents; breathing maneuvers
Duration	30 min; 60 min
Patient out-of-pocket cost	$500; $1000; $1500; $2000
Type of scanner	No scanner; Partial body scanner; Complete body scanner
Risks and side effects	Possible tingling in the fingers, dizziness and dry mouth; Possible chest pain, irregular heartbeat, flushing, and breathing difficulties and a 0.1% (1 in 1000) chance of serious complications such as heart attack; Exposure to radiation and a 0.1% (1 in 1000) increase in cancer risk
Diagnostic accuracy	90%; 80%; 70%

Six attributes were drawn to characterize cardiovascular imaging tests. Between two and four levels were applied to each attribute

The cost attribute was described as patient out-of-pocket cost, and all participants were instructed to answer as though patients had to pay for their imaging test, similar to uninsured services from a private clinic. It was estimated that non-invasive advanced cardiovascular imaging tests for diagnosing CAD would generally cost between $500 and $2000 [14]. Measures of specificity and sensitivity placed non-invasive cardiovascular imaging tests’ diagnostic accuracy ranging from approximately 70–90% [15]. Different procedures and modalities in cardiovascular imaging have been associated with a range of risks and side effects. Controlled hyperventilation followed by breath-holding was shown to induce myocardial vasomotion comparable to other stress test procedures [16]. Therefore, while being a new diagnostic test, the breathing maneuvers technique during CMR (b-CMR) was included along with exercise and pharmacological stress tests. Side-effects such as tingling in the fingers, dizziness and dry mouth have been reported during b-CMR [17]. Physical exercise and the use of pharmacological stress agents in CMR, ultrasound and nuclear medicine have both been associated with chest pain, palpitations, flushing, breathing difficulties, and a rare (0.1%) chance of serious complications such as myocardial infarction or death [18–20]. While patient-specific factors such as age and sex are important to consider, exposure to ionizing radiation resulting from nuclear medicine and CT modalities has been associated to an approximated 0.1% increase in the lifetime risks of developing cancer [21, 22]. Both groups were provided with the same attributes and levels to facilitate the comparison of their responses [23].

### Experimental model

A choice-based conjoint (CBC) design from Sawtooth software (https://www.sawtoothsoftware.com/products/conjoint-choice-analysis/cbc) was performed. A full access academic subscription license of the Sawtooth software (3210 N Canyon Road # 202, Provo UT, 84604-6508, United States of America) program was granted for this study.

Seven questionnaire versions of a Traditional Full-Profile CBC design were created, each containing 12 random pairwise choice scenarios (tasks) and two concepts per tasks, thus generating 84 unique choice tasks. In each questionnaire, one choice task was duplicated as a controlled fixed task, resulting in a total of 13 choice sets in all 7 questionnaires which were distributed consecutively to participants. Responses to the duplicated choice tasks were not included in the analysis. The model did not include an opt-out alternative, and no deviation from the experimental model was applied. Our two-alternative design yielded 648 possible profiles and produced 209 628 possible combinations of two-alternative choice questions. Selection of the profiles was performed with Sawtooth Software’s balanced overlap design. While there are no formal sample size calculations for discrete choice experiments, a common rule of thumb states that 20 participants per questionnaire version provides precise parameter estimates and reliable models [24]. With seven questionnaire versions, we therefore aimed at recruiting a minimum of 140 participants.

### Procedures

The study was conducted between November 2017 and May 2018 at the Research Institute of the McGill University Health Center (MUHC) in Montreal, Canada. All participants were adults over 18 years of age able to read and communicate in either English or French. Patients were recruited in-person in the waiting room of the MUHC’s Royal Victoria cardiology clinic where a researcher was available to speak the instructions of the experiment and answer questions. Attending cardiologists and family physicians affiliated with the MUHC were invited to participate in the study using a research participation invitation sent by a senior administrator through their hospital email. This study complied with the latest version of the Declaration of Helsinki and was carried out with approval of the local institutional ethics review board.

**Fig. 1:**
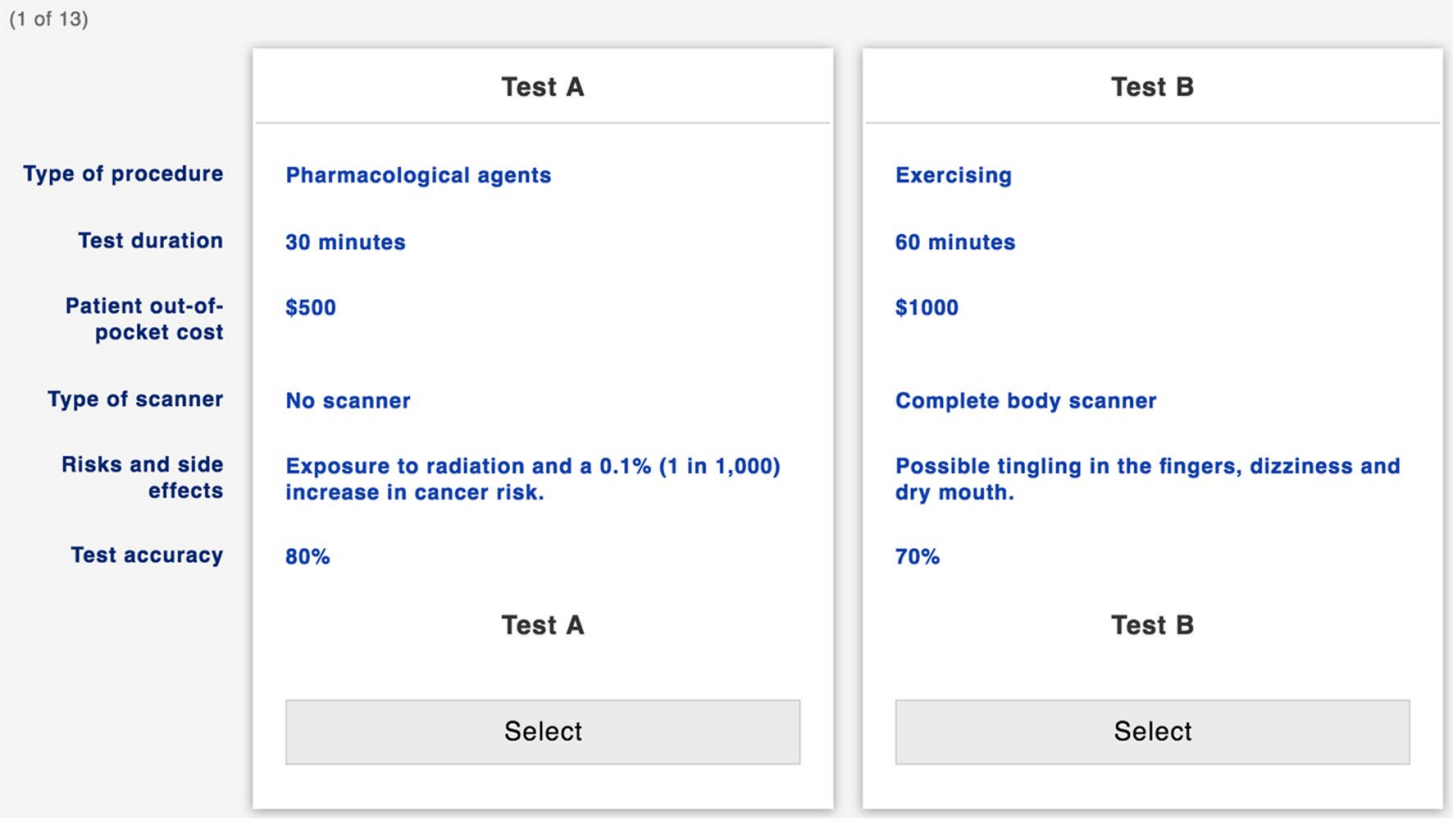
Example of a discrete choice task. *Note* Each discrete choice task shows the 6 test attributes on the left, and two hypothetical test alternatives (Test A and Test B) with their separate levels. Participants respond by selecting the test they prefer after considering each test characteristics

The online DCE questionnaire versions were accessible via the Sawtooth software's Hosting Services—Lighthouse Studio (https://www.sawtoothsoftware.com/products/online-surveys), from which paper-and-pencil copies were designed and made available in both English and French. Considering the minimal risks involved with participation, an implied consent form was presented to participants prior to enrolment. In the first part of the questionnaire, participants were asked to answer a demographic questionnaire and ordinal questions regarding their experience with different cardiovascular imaging tests. A detailed description of all attributes and levels was then presented to participants to ensure their understanding of the imaging test alternatives presented, followed by clear instructions on how to complete the DCE and the 13 choice tasks. Figure 1 shows an example of a discrete choice task. Following the DCE choice tasks, participants were questioned about their perception of the questionnaire difficulty and were instructed to submit their answers.

### Data analysis

A Sawtooth software’s CBC/Hierarchical Bayes (HB) analysis model was used to optimize the estimation of attributes and levels utilities, which were used to measure the relative importance, ranks and weights of each test attributes using 95% confidence intervals for significance testing. To facilitate the interpretation with respect to relative utilities variations, zero-centered utilities were calculated. A counting analysis was performed to obtain a concise ratio-scaled calculation of main effects, which indicated whether the various levels of an attribute differ significantly in their frequency of choice. The size and statistical significance of the main effects were calculated using within-attribute Chi-Squares. Measures of central tendencies and independent t-tests were computed using the Statistical Package for the Social Science (SPSS) Statistics for Macintosh, Version 22.0 (Released 2013, IBM Corp. Armonk, NY). A market simulation based on the CBC/HB utilities was conducted to uncover shares of preference for the seven existing imaging tests previously mentioned, to which each was assigned applicable levels (Table 2). Shares of preferences were estimated using a randomized first choice simulation method.

**Table 2:** Comparison of Test alternatives for market simulations

imaging test	Type of procedure	Test duration (minutes)	Patient out-of-pocket cost	Type of scanner	Risks and side effects	Diagnostic accuracy (%)
Stress perfusion CMR	Pharmacological agents	60	$1500	Complete body scanner	Moderate to severe	90
Exercise echo	Exercising	30	$500	No scanner	Moderate to severe	80
Pharmacological stress echo	Pharmacological agents	30	$500	No scanner	Moderate to severe	80
CTA	N/A	30	$500	Partial body scanner	Ionizing radiation	80
SPECT	Pharmacological agents	60	$1000	Partial body scanner	Moderate to severe; ionizing radiation	70
PET	Pharmacological agents	60	$2000	Partial body scanner	Moderate to severe; ionizing radiation	90
b-CMR	Breathing maneuvers	30	$1000	Complete body scanner	Mild	80

N/A; Not applicable, $; US dollars, Mild; Tingling in the fingers, dizziness and dry mouth, Moderate to severe; Chest pain, irregular heartbeat, flushing, breathing difficulties, and a 0.1% chance of serious complications, Ionizing radiation; Exposure to radiation and a 0.1% possible increase in cancer risk

*CMR* cardiovascular magnetic resonance, *CT* computed tomography, *SPECT* single photon emission computed tomography, *PET* positron emission tomography, *b-CMR* breathing maneuver-enhanced cardiovascular magnetic resonance

## Results

### Participants

A total of 211 consecutive participants, including 148 cardiac patients and 63 physicians (29 cardiologists and 34 family physicians) completed the questionnaire and were included in the analysis. Of the 211 eligible participants who returned the DCE questionnaire, 190 (90.0%) completed all the choice-based conjoint scenarios. Participants’ demographic characteristics, educational attainments, reported familiarity with cardiovascular imaging modalities, and perceived questionnaire difficulty are presented in Table 3.

**Table 3:** Demographic characteristics

	Patients (*n* = 148)	Physicians (*n* = 63)
	ƒ (%)	ƒ (%)
Age (*M* ± *SD*)	54.2 ± 18.2	49.0 ± 11.1
Gender (Male)	79 (53.7)	40 (63.5)
Language (English)	71 (48.0)	59 (93.7)
Education		
Elementary school	8 (5.4)	0 (0.0)
High school	48 (32.7)	0 (0.0)
College	33 (22.4)	0 (0.0)
University	51 (34.7)	63 (100)
Prefer not to answer	7 (4.8)	0 (0.0)
Familiarity with imaging modalities		
At least one modality	134 (91.2)	62 (98.4)
MRI	96 (65.3)	56 (88.9)
Echography	111 (75.5)	61 (96.8)
CT	44 (29.9)	56 (88.9)
SPECT	5 (3.4)	47 (74.6)
PET	14 (9.5)	43 (68.3)
Perceived questionnaire difficulty		
Very easy	24 (17.5)	10 (17.2)
Easy	56 (40.9)	18 (31.0)
Not easy, not difficult	51 (37.2)	25 (43.1)
Difficult	6 (4.4)	5 (8.6)
Very difficult	0 (0.0)	0 (0.0)

Participants answered either Male or Female, and either English or French

ƒ frequency, *M* Mean, *SD* standard deviation, MRI magnetic resonance imaging, CT computed tomography, SPECT single photon emission computed tomography, PET positron emission tomography

### Counting analysis

The counting analysis revealed statistically significant attribute main effects for risks and side effects, diagnostic accuracy, and patient out-of-pocket cost in both patients and physicians, indicating that levels of these attribute differed significantly in their frequency of choice (Table 4). The type of procedure attribute main effect reached statistical significance in the physician group only.

**Table 4:** Counting analysis of main effects

Test attributes	Main effects (χ^2^)	
	Patients	Physicians
Risks and side effects	157.70*	16.91*
Diagnostic accuracy	60.27*	25.56*
Patient out-of-pocket cost	4.29*	30.56*
Type of scanner	3.47	2.39
Type of procedure	3.45	10.30*
Duration	1.95	0.57

χ^2^ chi-squares. *Indicates statistical significance at the 0.05 level

### Hierarchical Bayes analysis

**Fig. 2:**
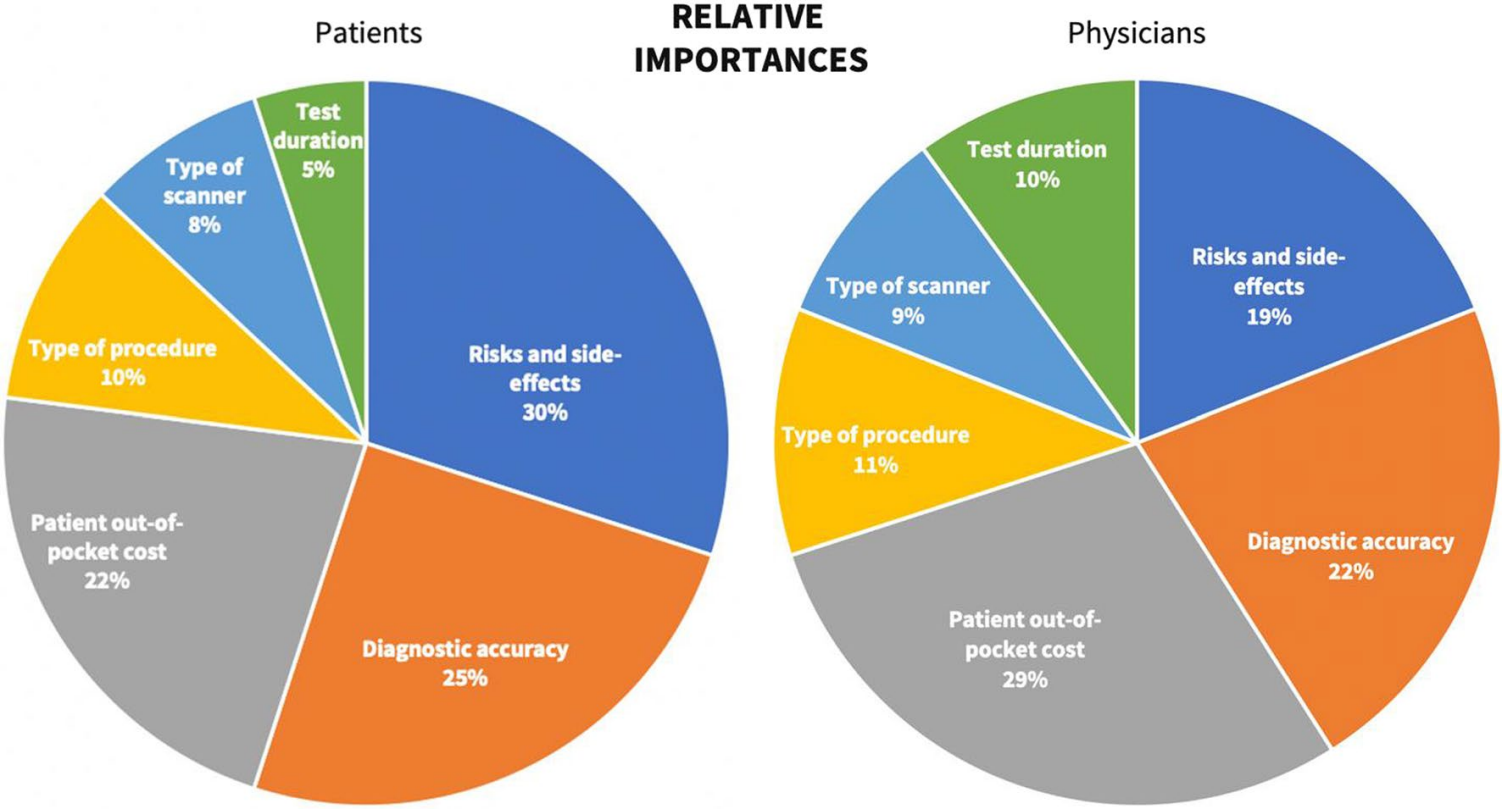
Hierarchical bayes analysis of test attributes’ relative importances according to patients and physicians. *Note* Relative importances were allocated to test attributes by patients (left) and by physicians (right). Each pie chart contains the six test attributes along with their respective relative preference value on a percentage scale

The CBC/HB analysis revealed diverging relative attribute importance and allocated zero-centered level utilities between patients and physicians. The relative importance of each cardiovascular test attribute is compared between participant groups and presented in Fig. 2.

**Fig. 3:**
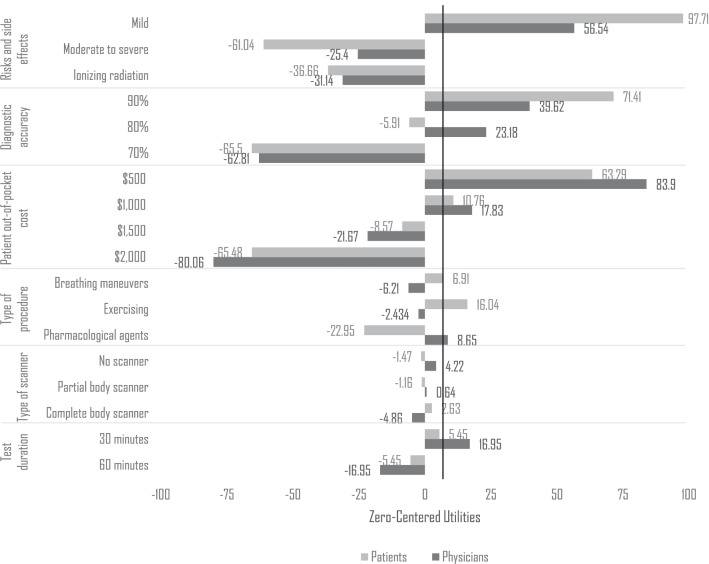
Hierarchical bayes zero-centered utilities. *Note* A higher and positive utility value denotes a higher preference, while a lower and negative value represents a lower preference relative to other test levels. Utilities for patients (dark grey) and by physicians (pale grey) appear laterally to their corresponding clustered bar. Abbreviations: Mild; Tingling in the fingers, dizziness and dry mouth, Moderate to severe; Chest pain, irregular heartbeat, flushing, breathing difficulties, and a 0.1% chance of serious complications, Ionizing radiation; Exposure to radiation and a 0.1% possible increase in cancer risk

Figure 3 details zero-centered utilities allocated to each attributes’ levels. Patients placed high utilities (+ 97.71) to tests with milder side effects such as tingling in the fingers, dizziness and dry mouth but avoided choosing tests involving exposure to ionizing radiation (− 36.66) and risks associated with exercise and the use of pharmacological agents inducing direct coronary arteriolar vasodilation (− 61.04).

**Fig. 4:**
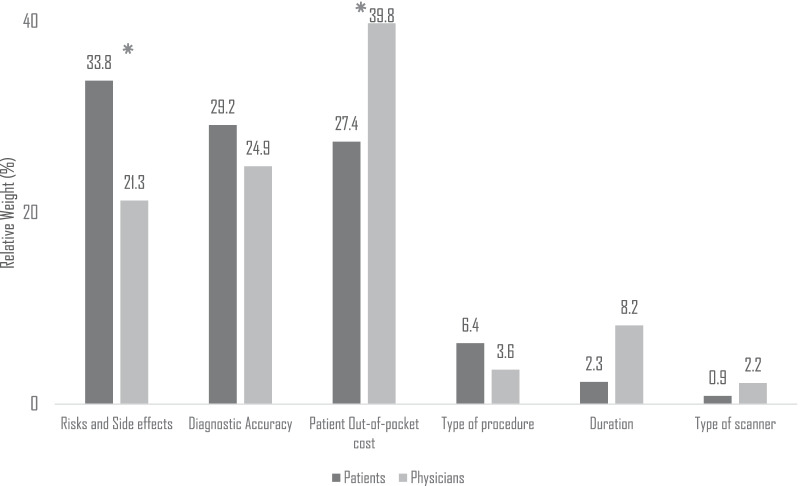
Comparison of test attribute relative weight in decision-making. *Note* Test attributes’ relative weight are expressed in percentages above their corresponding column by patients (dark grey) and by physicians (pale grey). *Indicates statistical significance at the 0.05 level

The attribute relative ranks and weights analysis revealed statistically significant differences between patient and physician preferences (Fig. 4). The risks and side effects attribute was ranked first by patients and third by physicians, and had significantly greater decision-making weight in the patient group (33.8% vs. 21.3%, *p* < 0.05).

### Market simulation

**Fig. 5:**
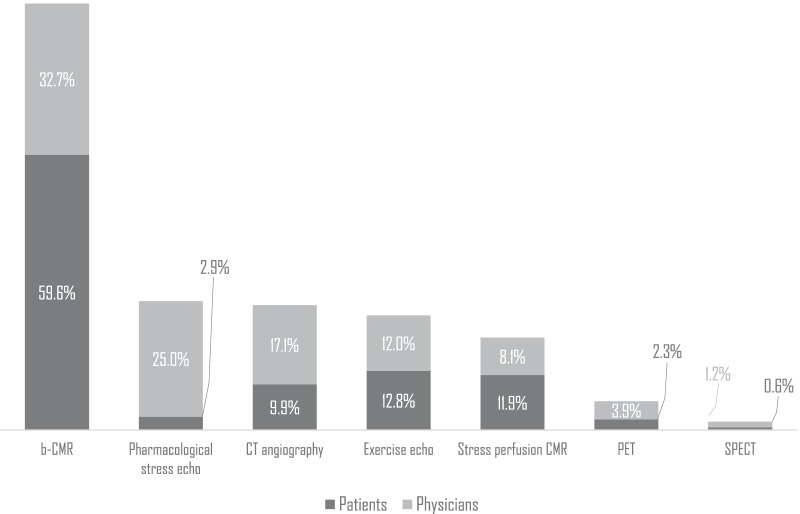
Shares of preferences for cardiovascular imaging tests. *Note* Shares of preferences by patients (bottom) and physicians (up) are expressed as percentages into stacked columns. Percentages appear in their corresponding column or are linked by callout lines. Higher percentages signify a greater likelihood the test will be chosen over other alternatives. *CMR* cardiovascular magnetic resonance, *CT* computed tomography, *SPECT* single photon emission computed tomography, *PET* positron emission tomography, *b-CMR* breathing maneuver-enhanced cardiovascular magnetic resonance

The application of the Hierarchical Bayes utilities to a market simulation comprised of the imaging tests alternatives described in Table 2 showed shares of preferences were highest for test features related to b-CMR in both the patient and the physician groups (Fig. 5). An estimated 59.6% of cardiac patients and 32.7% of physicians would prefer b-CMR over other diagnostic imaging tests.

## Discussion

This study used a discrete choice experiment to estimate patients’ and physicians’ preferences for attributes of non-invasive cardiovascular tests and imaging modalities used for diagnosing coronary heart disease and to simulate the value of combined imaging test features. Similar to most studies comparing patient and physician preferences in a DCE [23], our results showed mixed concordance and discordance between groups. Patients and physicians generally agreed on the importance of risks and side effects, diagnostic accuracy and patient out-of-pocket cost relative to other attributes, but their attribute preference raking differed.

When given the choice, the patients’ preference of specific cardiovascular imaging tests was mostly determined by the associated risks and side-effects. Specifically, patients preferred tests with mild side effects such as tingling in the fingers, dizziness and dry mouth, but avoided test alternatives with exposure to ionizing radiation and risks associated with exercise and the use of pharmacological agents inducing direct coronary arteriolar vasodilation. Conversely, physicians mostly valued tests of lower cost to their patients. In many ways, these findings support the American College of Cardiology’s recommendations toward creating an accountability framework to more safely drive appropriate imaging utilization [25], and compare favorably with previous DCE studies showing that patients value medical interventions with minimized side effects [26, 27]. Our results were also in line with a DCE reporting that economic considerations played an important role in physicians’ treatment decisions [28]. The discordance of importance assigned to the risks and side effects attribute between patients and physicians may be rationalized by the greater perceived risks associated with diagnostic tests in patients. For trained physicians commonly ordering diagnostic tests, a 0.1% chance of complications may seem benign when compared to the risks associated with more invasive procedures. Our results offer an analysis of the concordance and discordance between patients’ and physicians’ preference for cardiovascular imaging tests. Considering that physicians order tests for their patients, and that their patients’ preference should be accounted for within a shared-decision making framework, physicians and policymakers should reflect on these discordances and orient their professional judgement towards patient-preferred alternatives.

While this study did not dispute the patient-centeredness of other cardiovascular imaging tests and modalities, the simulation revealed the b-CMR test had the highest shares of preferences in both patients and physicians. This could be explained because b-CMR was associated with mild risks and side-effects, which was allocated the highest utilities by patients. b-CMR did not have the preferred levels for the patient out-of-pocket cost and diagnostic accuracy attributes, but physicians also found its overall features more appealing than other imaging test alternatives.

### Limitations

This study was performed in a Canadian environment, and it would, therefore, be challenging to estimate how these results would apply to other countries with dissimilar healthcare systems. In addition, because the Canadian publicly funded government health insurance covers most medical interventions in Canada, the cost attribute was not completely representative of the choice patients would have to make in this context. DCE results are only as precise as the attributes and levels used to describe the services of interest. While most important imaging tests’ attributes used in this study were determined through literature review and interviews with key informants in the context of cardiology, these methods did not guarantee that all tests’ attributes relevant to the existing imaging tests were included. Having added more test attributes and levels might have provided more precise information to characterize non-invasive cardiovascular imaging tests for diagnosing CAD but could have counterproductively made the experiment more cognitively demanding for participants [29]. Of note, only 4.4% of patients and 8.6% of physicians found the questionnaire difficult, and none of the participants reported the questionnaire as being very difficult, which suggests errors related to task unclarity and excessive cognitive demand were minimal.

### Conclusions

When given the choice, patients preference for non-invasive cardiovascular imaging tests for coronary artery disease was most determined by associated risks and side effects, more than by diagnostic accuracy and cost-efficiency. In accordance with several published recommendations for patient-centered care, cardiovascular imaging test selection should be subject to shared decision-making, with special emphasis on associated risks and side effects.

## Data Availability

The datasets used and/or analysed during the current study are available from the corresponding author on reasonable request.
